# Safety and efficacy of ferric citrate in patients with nondialysis-dependent chronic kidney disease

**DOI:** 10.1371/journal.pone.0188712

**Published:** 2017-11-29

**Authors:** Glenn M. Chertow, Geoffrey A. Block, John F. Neylan, Pablo E. Pergola, Katrin Uhlig, Steven Fishbane

**Affiliations:** 1 Stanford University School of Medicine, Department of Medicine - Med/Nephrology, Stanford, California, United States of America; 2 Denver Nephrology, Denver, Colorado, United States of America; 3 Keryx Biopharmaceuticals, Inc., Boston, Massachusetts, United States of America; 4 Renal Associates PA, San Antonio, Texas, United States of America; 5 Hofstra Northwell School of Medicine, Division of Medicine - Kidney Diseases and Hypertension, Great Neck, New York, United States of America; Universidade Nove de Julho, BRAZIL

## Abstract

Two randomized, placebo-controlled trials conducted in patients with nondialysis-dependent (NDD) chronic kidney disease (CKD), iron deficiency anemia, and normal or elevated serum phosphorus demonstrated that ferric citrate (FC) significantly increased hemoglobin and decreased serum phosphate concentrations. Pooling these trial results could provide a more robust evaluation of the safety and efficacy of FC in this population. We pooled results of a phase 2 (n = 149) and 3 trial (n = 233) of patients randomized and treated for up to 12 and 16 weeks, respectively. The starting dose in both trials was three 1-g (elemental iron 210 mg) tablets/day with food, up to 12 tablets/day. Doses were titrated in the phase 2 and 3 trials to lower serum phosphate concentrations to a target range (0.97–1.13 mmol/L) and to achieve a ≥10-g/L hemoglobin increase, respectively. Safety was assessed in all patients who received ≥1 dose of FC (n = 190) and placebo (n = 188). Treatment-emergent adverse events (AEs) were reported in 143 of 190 (75.3%) FC-treated and 116 of 188 (61.7%) placebo-treated patients; gastrointestinal AEs were the most frequent (94 [49.5%] vs. 52 [27.7%], respectively). Specific events reported in >5% of patients (FC vs. placebo, respectively) included discolored feces (41 [21.6%] vs. 0 [0.0%]), diarrhea (39 [20.5%] vs. 23 [12.2%]), constipation (35 [18.4%] vs. 19 [10.1%]), and nausea (18 [9.5%] vs. 8 [4.3%]). Twenty FC-treated (10.5%) and 21 placebo-treated patients (11.2%) experienced a serious AE. Two patients (1.1%) died in each group. A pooled efficacy assessment demonstrated a consistent hemoglobin rise and modest serum phosphate decline, with few excursions below the normal range. When used for treatment of patients with NDD-CKD, FC contributes to gastrointestinal AEs at higher rates than placebo, while simultaneously correcting two of the principal metabolic manifestations of CKD (iron deficiency anemia and relative hyperphosphatemia).

## Introduction

Chronic kidney disease (CKD), as currently defined, is reported to be present in one in seven individuals in the United States (US) [1]. Although >650,000 patients require either dialysis or a functioning kidney transplant to sustain life, the vast majority of patients with CKD do not. A heightened risk of cardiovascular disease may be the most significant complication of moderate to advanced CKD [2,3]; other clinical manifestations can include impaired physical and cognitive function, frailty, protein energy wasting, fractures [4–7], and numerous laboratory abnormalities including iron deficiency and hyperphosphatemia. Iron deficiency [8–10] contributes to anemia in CKD [11], as well as to heart failure [12,13], central nervous system dysfunction, and diminished health-related quality of life in some patients [14,15]. Hyperphosphatemia can contribute to dystrophic calcification, secondary hyperparathyroidism, and other abnormalities of mineral metabolism [16].

The optimal management strategies for iron deficiency and phosphate retention in advanced, nondialysis-dependent CKD (NDD-CKD) are unknown. Ferric citrate was initially developed as a phosphate binder and received regulatory approval for the control of serum phosphorus in patients with dialysis-dependent CKD (DD-CKD) [17]. When prescribed for the treatment of hyperphosphatemia in DD-CKD, investigators observed that ferric citrate not only decreased serum phosphate concentrations but also increased transferrin saturation (TSAT) and serum ferritin concentrations, allowing patients to maintain hemoglobin concentrations on lower doses of erythropoiesis-stimulating agents (ESAs) and intravenous iron [18]. Given the expanding evidence base linking higher serum phosphate concentrations to adverse events (AEs) in individuals with impaired as well as normal or near-normal kidney function, we initially designed a phase 2 placebo-controlled trial (ClinicalTrials.gov identifier: NCT01736397) to examine the effects of ferric citrate among patients with NDD-CKD, iron deficiency anemia, and high-normal or elevated serum phosphate (“relative hyperphosphatemia”) [19]. Having observed favorable effects on hemoglobin, TSAT, and ferritin levels with ferric citrate dosing guided by serum phosphate concentrations, we designed a phase 3 placebo-controlled trial (NCT02268994) to examine the effects of ferric citrate among patients with iron deficiency anemia with dosing guided by hemoglobin concentrations [20]. Primary results of these trials have been previously published [20]. Herein, we pool results from both trials, primarily to obtain a more robust assessment of the safety of ferric citrate. Since both trials included similar populations, doses, and duration and captured the same laboratory-based outcomes, we also pool results to determine efficacy.

## Materials and methods

### Design of the phase 2 and 3 studies

The phase 2 study was a 12-week, randomized, multicenter, double-blind, placebo-controlled trial undertaken at 27 centers in the US, with the first patient visit in November 2012 and last patient visit in October 2013. The phase 3 study was a randomized, multicenter, double-blind, placebo-controlled trial comprised of a 16-week randomized period followed by an 8-week safety extension period and undertaken at 32 centers in the US, with the first patient visit in October 2014 and last patient visit in January 2016. Both trials were conducted in accordance with Title 21, US Code of Federal Regulations Parts 11, 50, 54, 56, and 312; the International Conference on Harmonisation guideline on Good Clinical Practices (E6, April 1996); the Declaration of Helsinki (Seoul, October 2008); and applicable local regulatory requirements and laws. All versions of the protocol implemented in each study were reviewed and approved by the Liberty Institutional Review Board before patient enrollment at each site. Institutional review board-approved written informed consent was obtained from each patient before performing any study assessments. Detailed methods for both trials have been previously published [19,20].

### Study population

Patients in both trials had CKD stages 3–5 (estimated glomerular filtration rate <60 mL/min/1.73 m^2^) and were not receiving dialysis. In the phase 3 trial, patients had intolerance or an inadequate response to conventional oral iron formulations; this was not an eligibility criterion in the phase 2 trial. In the phase 2 trial, patients were required to have serum phosphate ≥1.29 and <1.94 mmol/L at baseline, whereas serum phosphate was required to be ≥1.13 mmol/L in the phase 3 trial. Inclusion criteria regarding iron deficiency anemia differed slightly otherwise: screening TSAT, ferritin, and hemoglobin concentrations were required to be <30%, <674 pmol/L, and 91–119 g/L, respectively, in the phase 2 trial; corresponding values were <25%, <449 pmol/L, and 90–115 g/L in the phase 3 trial. Phase 3 trial participants tended to have slightly more pronounced iron deficiency and anemia, although there was extensive overlap across the two trial samples vis-à-vis baseline serum phosphate, hemoglobin, TSAT, and ferritin. Neither intravenous iron nor ESAs were allowed during the 12-week or 16-week placebo-controlled treatment periods in the phase 2 and 3 trials, respectively. The starting dose in both trials was three 1-g (210 mg elemental iron) tablets/day with food, which could be increased to a maximum of 12 tablets/day. In the phase 2 trial, the dose was titrated to achieve a target serum phosphate concentration (0.97–1.13 mmol/L); in the phase 3 trial, the dose was titrated to achieve a ≥10-g/L increase in hemoglobin.

#### Analysis populations

The pooled safety analysis included all patients who received ≥1 dose of study drug (ferric citrate or placebo) and data from baseline until the end of the randomized period of each study (12 weeks for the phase 2 trial; 16 weeks for the phase 3 trial). The pooled efficacy analysis included all patients who were randomized, received ≥1 dose of study drug, and had ≥1 postbaseline laboratory value in the randomized period. Nine patients were enrolled in both trials; of these, three were randomized to the same treatment in both trials and only data from the phase 3 trial were included for these patients. Six patients were randomized to receive different treatments in the phase 2 and 3 trials, and these six patients contributed data from each trial.

### Safety and efficacy end points

Key safety end points included all treatment-emergent AEs and serious AEs, as traditionally defined, at any point up to the end of the randomized period of each trial. Based on prior trials of ferric citrate in DD-CKD, we considered gastrointestinal AEs and ferritin ≥1573 pmol/L, TSAT ≥70%, and serum phosphate <0.65 mmol/L as outcomes of special interest.

Efficacy end points included those related to the treatment of iron deficiency anemia: the proportion of patients achieving a ≥10-g/L increase in hemoglobin at any time during the randomized study periods and mean changes in hemoglobin, TSAT, ferritin, and serum phosphate from baseline to week 12.

### Statistical analysis

For safety data, we provide counts and proportions. Inference tests were not performed. For the analysis of discrete proportions, we used the Cochran-Mantel-Haenszel test stratified by trial to estimate the difference in the proportion of patients randomized to ferric citrate and placebo who achieved a ≥10-g/L increase in hemoglobin at any point up to the end of each trial. We performed a Breslow-Day test for homogeneity to assess whether the effect of treatment was similar in the phase 2 and 3 trials using a significance level of *P* = 0.10. We analyzed changes from baseline in laboratory values to all postbaseline time points during randomized treatment up to week 12 with a mixed model for repeated measures (MMRM) using restricted maximum likelihood with treatment (ferric citrate and placebo), trial (phase 2 and phase 3), visit, and treatment-by-visit interaction as fixed effects; the baseline variable as a covariate; and patient as a random effect. Finally, we added a term for trial-by-treatment interaction to the model to assess whether the effect of treatment differed by trial, which was tested at a significance level of *P* = 0.10. A compound symmetry covariance structure was used to model within-patient errors. Point estimates and 95% confidence intervals for the treatment differences at each assessment time, along with *P* values for the treatment comparisons, were obtained from the model. There was no imputation of missing data. We conducted all analyses using SAS version 9.4 (SAS Institute Inc., Cary, NC, USA).

## Results

**Fig 1:**
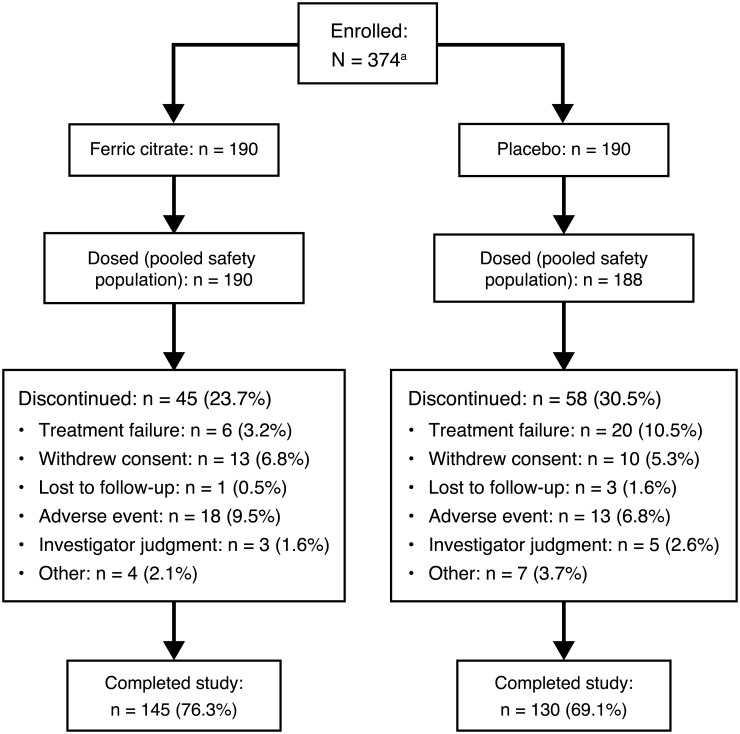
CONSORT diagram. ^a^Nine patients were enrolled in both studies; of these, three were randomized to the same treatment in both trials and only their data from the phase 3 trial were included, and six patients who were randomized to different treatments contributed data from each trial but were only counted once in the total number of enrolled subjects.

Fig 1 shows the disposition of patients from the two trials. The pooled safety population included 190 ferric citrate-treated and 188 placebo-treated patients; of these, 145 (76.3%) and 130 (69.1%) patients, respectively, completed the randomization period. Baseline characteristics of all participants are shown in Table 1.

**Table 1:** Baseline characteristics (pooled data).

Characteristic	Ferric citrate (n = 190)	Placebo (n = 188)
Age, years		
Mean (SD)	65.5 (11.6)	64.8 (13.3)
Min–max	25–87	21–93
Sex, n (%)		
Female	126 (66.3)	115 (61.2)
Race, n (%)		
White	135 (71.1)	137 (72.9)
Black	54 (28.4)	47 (25.0)
Other	1 (0.5)	4 (2.1)
Ethnicity, n (%)		
Hispanic	43 (22.6)	45 (23.9)
Weight, kg		
Mean (SD)	92.6 (22.7)	91.1 (24.1)
Min–max	46.9–154.7	47.9–167.8
Past medical history, n (%)		
Diabetes mellitus	132 (69.5)	131 (69.7)
Heart failure	40 (21.1)	41 (21.8)
Ischemic heart disease	53 (27.9)	55 (29.3)
Current stage of CKD, n (%)		
Stage 3	78 (41.1)	87 (46.3)
Stage 4	88 (46.3)	83 (44.1)
Stage 5	24 (12.6)	18 (9.6)
eGFR, mL/min/1.73 m^2^		
Mean (SD)	26.7 (12.5)	26.8 (11.9)
Min–max	6–66	8–63
Hemoglobin, g/dL		
Mean (SD)	10.48 (0.76)	10.47 (0.90)
Min–max	8.5–12.4	8.4–14.7
Transferrin saturation, %		
Mean (SD)	20.9 (6.8)	20.3 (7.4)
Min–max	6–50	4–45
Ferritin, ng/mL		
Mean (SD)	98.0 (68.9)	92.5 (68.5)
Min–max	3–380	6–355
Serum phosphate, mg/dL		
Mean (SD)	4.34 (0.81)	4.33 (0.70)
Min–max	2.8–8.7	2.8–6.9
Bicarbonate, mmol/L		
Mean (SD)	21.0 (3.6)	21.6 (3.6)
Min–max	10–30	12–30

To convert g/dL to g/L for hemoglobin, multiply by 10. To convert ng/mL to pmol/L for ferritin, multiply by 2.247. To convert mg/dL to mmol/L for phosphate, multiply by 0.323.

CKD, chronic kidney disease; eGFR, estimated glomerular filtration rate; SD, standard deviation.

### Treatment duration and dose

The mean (standard deviation [SD]) treatment durations in the ferric citrate and placebo groups were 12.8 (4.2) and 12.1 (4.9) weeks, respectively, with corresponding mean (SD) study drug doses of 5.4 (1.5) and 5.7 (1.7) g/day.

### Safety

**Fig 2:**
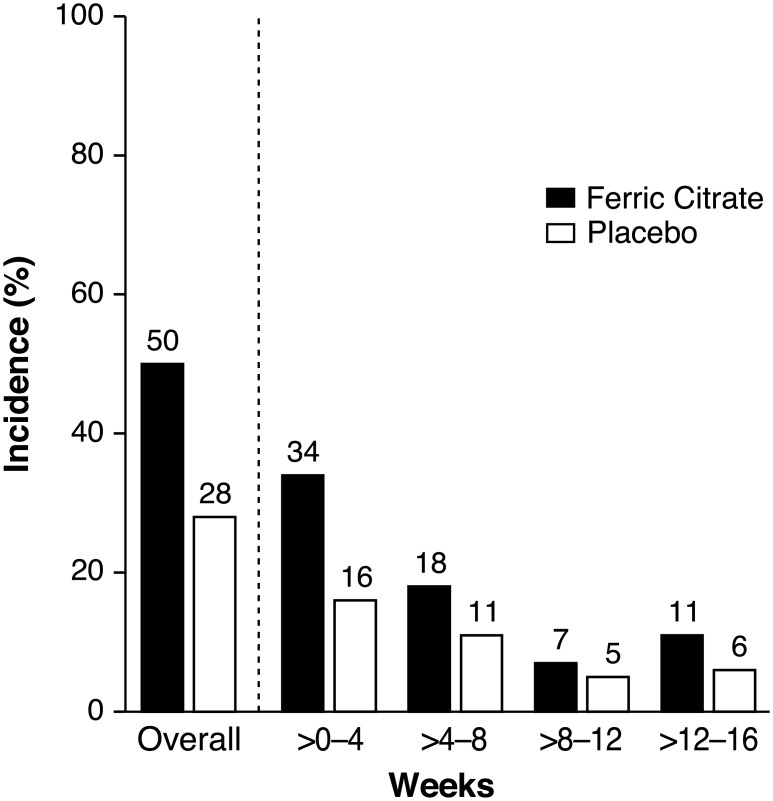
Gastrointestinal adverse events by study week. Discontinuations because of gastrointestinal treatment-emergent adverse events during the randomized periods (ferric citrate [5.3%] vs. placebo [1.1%]).

Treatment-emergent AEs were reported in 143 ferric citrate-treated (75.3%) and 116 placebo-treated patients (61.7%); gastrointestinal AEs were the most frequent (94 [49.5%] vs. 52 [27.7%], respectively). Ten ferric citrate-treated patients (5.3%) and two placebo-treated patients (1.1%) discontinued the study drug because of gastrointestinal AEs. Specific events reported in >5% of patients (ferric citrate vs. placebo, respectively) included discolored feces (41 [21.6%] vs. 0 [0.0%]), diarrhea (39 [20.5%] vs. 23 [12.2%]), constipation (35 [18.4%] vs. 19 [10.1%]), and nausea (18 [9.5%] vs. 8 [4.3%]). Twenty ferric citrate-treated patients (10.5%) and 21 placebo-treated patients (11.2%) experienced a serious AE; the highest proportion of serious AEs were cardiac disorders (3.7% vs. 2.7%, respectively) and infections and infestations (2.6% vs. 3.7%, respectively). Fig 2 describes gastrointestinal AEs by study week. Table 2 includes additional, less commonly observed gastrointestinal AEs. Two patients (1.1%) died in each treatment group. None of the serious AEs or deaths were deemed to be related to the study drug.

**Table 2:** Gastrointestinal AEs observed in ≥2% of patients.

Gastrointestinal AEs, n (%)	Ferric citrate (n = 190)	Placebo (n = 188)
Any AE (≥2% of patients)	94 (49.5)	52 (27.7)
Feces discolored	41 (21.6)	0 (0.0)
Diarrhea	39 (20.5)	23 (12.2)
Constipation	35 (18.4)	19 (10.1)
Nausea	18 (9.5)	8 (4.3)
Abdominal pain	9 (4.7)	3 (1.6)
Vomiting	8 (4.2)	8 (4.3)
Flatulence	6 (3.2)	3 (1.6)
Abdominal discomfort	4 (2.1)	1 (0.5)
Dyspepsia	4 (2.1)	1 (0.5)

AE, adverse event.

Episodes of TSAT ≥70%, serum ferritin ≥1573 pmol/L, and serum phosphate <0.65 mmol/L were observed in 28 (14.9%), one (0.5%), and two (1.1%) ferric citrate-treated patients, respectively, compared with 0 (0.0%), 0 (0.0%), and 0 (0.0%) placebo-treated patients, respectively.

### Efficacy

#### Iron deficiency anemia

**Fig 3:**
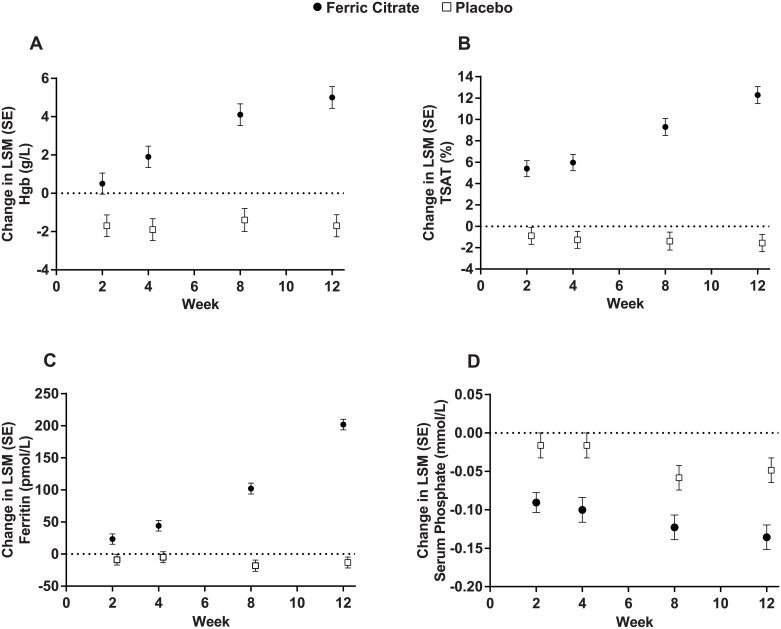
Pooled changes from baseline in (A) Hgb, (B) TSAT, (C) ferritin, and (D) serum phosphate over the 12-week randomized treatment period in the phase 2 trial and the first 12 of 16 weeks of the randomized treatment period in the phase 3 trial. Changes were analyzed using mixed model for repeated measures with restricted maximum likelihood, with treatment (ferric citrate vs. placebo), study, visit, and treatment-by-visit interaction as fixed effects; baseline as a covariate; and patient as a random effect. Study-by-treatment interaction *P* value was obtained from the same model with the addition of the study-by-treatment interaction term. Hgb, hemoglobin; LSM, least-squares mean; SE, standard error; TSAT, transferrin saturation.

The proportion of patients who achieved a ≥10-g/L increase in hemoglobin from baseline at any time during the randomization period was higher among patients treated with ferric citrate compared with placebo (47.8% vs. 18.6%, respectively; difference of proportions, 28.7%; *P* < 0.001. Breslow-Day test for homogeneity indicated no differences across trials, *P* = 0.46. At week 12, the mean (SD) change in hemoglobin was 5.0 (0.6) g/L in the ferric citrate group versus –1.7 (0.6) g/L in the placebo group (mean difference, 6.7 g/L; *P* < 0.001). The mean (SD) change in TSAT was 12.3 (0.80) % in the ferric citrate group versus –1.6 (0.81) % in the placebo group (mean difference, 13.8%; *P* < 0.001). The mean (SD) change in ferritin was 210.8 (8.5) pmol/L in the ferric citrate group versus –13.3 (8.5) pmol/L in the placebo group (mean difference, 224.0 pmol/L; *P* < 0.001). Fig 3A–3C show pooled results for hemoglobin, TSAT, and ferritin over the 12-week randomized treatment period in the phase 2 trial and for the first 12 of 16 weeks of the randomized treatment period in the phase 3 trial. The trial-by-treatment interaction showed no treatment heterogeneity between trials for hemoglobin (*P* = 0.62) but suggested heterogeneity for TSAT and ferritin (*P* = 0.055 and *P* = 0.076, respectively). TSAT and ferritin increased in ferric citrate-treated patients in both trials, with more pronounced treatment effects observed in the phase 3 trial.

#### Phosphorus

At week 12, the mean (SD) change in serum phosphate was –0.14 (0.02) mmol/L in the ferric citrate group versus –0.05 (0.02) mmol/L in the placebo group (mean difference, –0.09 mmol/L; *P* < 0.001). There was a reduction in serum phosphate in ferric citrate-treated patients in both trials, with more pronounced treatment effects observed in the phase 2 trial (*P* < 0.001). Fig 3D shows pooled results for serum phosphate over the 12-week randomized treatment period in the phase 2 trial and for the first 12 of 16 weeks of the randomized treatment period in the phase 3 trial.

## Discussion

This pooled analysis of two randomized, placebo-controlled trials, which comprises the largest dataset of patients with NDD-CKD stages 3–5 treated with ferric citrate to date, has enabled a comprehensive evaluation of potential AEs with ferric citrate, including hypophosphatemia, in patients with NDD-CKD with normal to high phosphate levels and iron deficiency anemia. Of note, no major or unexpected safety concerns were observed in this analysis. We also report a more complete and detailed evaluation of gastrointestinal AEs compared with prior publications of the individual trials. We show that gastrointestinal AEs including diarrhea, constipation, and nausea were relatively common, but few were classified as serious or resulted in hospitalization or discontinuation of medication. The frequency of AEs among placebo-treated patients in CKD trials is typically high, but the percentage of ferric citrate-treated patients who experienced AEs was greater by 14% for any treatment-emergent AE, 22% for any gastrointestinal AE, 8% for diarrhea, 8% for constipation, 5% for nausea, and 3% for abdominal pain. There have been no head-to-head comparisons of ferric citrate and other iron formulations. Bearing that in mind, the AEs for ferric citrate in the pooled dataset are similar to those in other clinical trials in which conventional oral iron formulations (e.g. ferrous sulfate, fumarate, and gluconate) have been used [21–23] and to clinical practice experience, where AEs (predominantly constipation) of conventional oral iron formulations are common [22]. The discontinuation rate due to gastrointestinal AEs in this trial (5.3%) was in line with that observed with other conventional oral iron formulations; however, it is difficult to make a direct comparison across trials because of variations in the methodology of AE reporting, particularly with respect to how discontinuations due to AEs were reported [24–26].

While these trials did not compare ferric citrate to conventional oral iron formulations or intravenous iron in a head-to-head manner, ferric citrate showed robust efficacy in light of the effects seen with conventional oral iron formulations in other trials in this patient population, although patients in the ferric citrate trials were less iron deficient at baseline [24,25,27,28]. Similarly, although ferric citrate has not been compared directly with other phosphate binders in this patient population, the achieved decrease in serum phosphate was larger than in another randomized, placebo-controlled trial in patients with CKD stages 3–5 in which calcium acetate, sevelamer carbonate, and lanthanum carbonate were compared [29]. Trials with head-to-head comparisons would enable more definitive conclusions to be made on the relative efficacy of ferric citrate. A trial comparing ferric citrate versus ferrous sulfate on iron parameters and hemoglobin in patients with CKD and iron deficiency is currently underway (NCT02888171).

It is noteworthy that the incidence of all gastrointestinal AEs declined over time despite an increase in the study medication dose. This phenomenon could be due to patients developing awareness of stool discoloration when using oral iron salts and/or adjusting to an increase in the number and frequency of bowel movements. These results should serve as a reminder to health care providers to inform patients starting ferric citrate of possible expectations regarding changes in stool color and consistency or frequency of bowel movements. Patients should also be reminded that, once on therapy, subsequent changes in stool color or character should prompt discussion with health care providers, so that other gastrointestinal issues (e.g. melena or hematochezia) are not masked.

Treatment with ferric citrate in this patient population produced a modest decline in serum phosphate that generally stayed within the normal range. It was reassuring that the incidence of hypophosphatemia was low (serum phosphate concentrations did not go lower than 1.6 mg/dL/0.09 mmol/L); however, it should be noted that patients with low-normal serum phosphate concentrations were excluded from participation. There remains some debate on the use of phosphate binders in patients with moderate to advanced CKD and normal or near-normal serum phosphate. A pilot 4-arm trial (calcium acetate, sevelamer carbonate, lanthanum carbonate and placebo) showed only modest reductions in serum phosphate and failed to attenuate vascular calcification [29]. In view of potential adverse skeletal and extraskeletal effects of prolonged hypophosphatemia, physicians may consider periodic monitoring of patients receiving ferric citrate for the treatment of iron deficiency anemia with low or low-normal serum phosphate concentrations, and severe nutritional vitamin D deficiency should be corrected. It is now recognized that 1,25-dihydroxy vitamin D deficiency in patients with NDD-CKD can be exacerbated by sustained increases in fibroblast growth factor 23 (FGF23), which directly suppress cytochrome P450 (CYP)27 (1-alpha-hydroxylase) and stimulate catabolic CYP24 (24-hydroxlase) [30–32]. Another interesting effect of ferric citrate is the reduction of FGF23 [19,20]. Elevated FGF23 in patients with CKD has been shown to be a risk factor for left ventricular hypertrophy and cardiovascular events, progressive loss of kidney function, and death [33–37].

A sizeable fraction of patients treated with ferric citrate experienced a transient increase in TSAT levels above 70%, although these elevated levels were not sustained. We believe that the transient increases in TSAT reflect the fact that the blood draws were not timed relative to drug intake. As described by Kobune et al., TSAT levels can rise above 60% within 2–3 hours after intake of an oral iron dose [38]. In future trials, it will be important to specify that laboratory draws for TSAT be obtained before daily dosing to minimize lability following oral ingestion of iron-containing compounds. As per the US Food and Drug Administration label, ferric citrate is contraindicated in patients with iron overload syndromes [17], and patients with a history of hemochromatosis were excluded from participating in these clinical trials.

Two additional studies examined the provision of ferric citrate in patients with NDD-CKD but were not similar enough to be included in the pooling: a Japanese trial of patients with hyperphosphatemia in which ferric citrate was used as a phosphate binder but in a lower dose and with concomitant use of intravenous iron and ESAs [39], and another small trial (NCT02128074) without a placebo control in which ferric citrate was given at a lower dose without food. The adverse event profiles in these studies were similar to that reported here [39].

This pooled analysis provides the most robust results available to date regarding the safety of ferric citrate when used for the treatment of either iron deficiency anemia or relative hyperphosphatemia in patients with NDD-CKD. Major limitations include the relatively small sample size and short duration of treatment. Also, women were overrepresented in the trials, most likely due to the higher prevalence of iron deficiency anemia in women than in men. Trial participants were more likely to be obese by Quételet’s (body mass) index than what might be expected for a typical population with iron deficiency anemia and normal or near normal kidney function, probably related to the higher prevalence of, and rates of progression with, CKD in the setting of obesity [1].

Ferric citrate is approved for the treatment of hyperphosphatemia in adult patients receiving dialysis and as an iron replacement product for the treatment of iron deficiency anemia in adult patients with chronic kidney disease not on dialysis. It will be important to assess longer-term safety end points in clinical practice and to determine its optimal use for maintenance therapy. The recently initiated COMPASS trial (NCT03236246) is designed to assess long-term safety and efficacy (over 48 weeks) of ferric citrate in patients with NDD-CKD and iron deficiency anemia.

## Conclusions

In summary, results from a pooled analysis of two randomized, placebo-controlled trials of ferric citrate for iron deficiency anemia with or without relative hyperphosphatemia in patients with NDD-CKD show a modest increase in diarrhea, constipation, nausea, and other nonserious gastrointestinal signs and symptoms, with no major or unexpected safety signals. Beneficial effects on the treatment of iron deficiency anemia and bone and mineral metabolism were also observed. Longer-term trials and observational studies examining the effects of ferric citrate on these and other intermediate outcomes, as well as on mortality, cardiovascular events, and fractures, are warranted.

## Supporting information

S1 Table Number and percent of subjects in the pooled safety population with treatment-emergent gastrointestinal adverse events by system organ class and preferred term. (PDF)

S2 Table Number and percent of subjects in the pooled safety population with treatment-emergent gastrointestinal adverse events by time to onset. (PDF)

S3 Table Mixed model repeat measures of change in hemoglobin from baseline over time during the double-blind period in the pooled safety population. (PDF)

S4 Table Mixed model repeat measures of change in iron parameters from baseline over time during the double-blind period in the pooled safety population. (PDF)

S5 Table Mixed model repeat measures of change in phosphate from baseline over time during the double-blind period in the pooled safety population. (PDF)
