# Supplementary material for: Safety and efficacy of ferric citrate in patients with nondialysis-dependent chronic kidney disease
Source: PLoS One. 2017 Nov 29;12(11):e0188712. doi: 10.1371/journal.pone.0188712 (PMC5706696; doi:10.1371/journal.pone.0188712)
Supplement: S1 Table — (PDF) [file pone.0188712.s001.pdf]

Table 3.22.1
Number and Percent of Subjects with Treatment-Emergent Gastrointestinal (GI) Adverse Events
by System Organ Class and Preferred Term
Pooled Safety Population

System Organ Class<br>Preferred Term	All<br>KRX-0502 [1]<br>(N=301)<br>n(%)	Double-Blind<br>KRX-0502<br>(N=190)<br>n(%)	Double-Blind<br>Placebo<br>(N=188)<br>n(%)
Any Treatment-Emergent GI AEs [2]	137 (45.5)	94 (49.5)	52 (27.7)
GASTROINTESTINAL DISORDERS	137 (45.5)	94 (49.5)	52 (27.7)
FAECES DISCOLOURED	59 (19.6)	41 (21.6)	0
DIARRHOEA	57 (18.9)	39 (20.5)	23 (12.2)
CONSTIPATION	43 (14.3)	35 (18.4)	19 (10.1)
NAUSEA	22 (7.3)	18 (9.5)	8 (4.3)
ABDOMINAL PAIN	15 (5.0)	9 (4.7)	3 (1.6)
VOMITING	10 (3.3)	8 (4.2)	8 (4.3)
ABDOMINAL DISCOMFORT	8 (2.7)	4 (2.1)	1 (0.5)
FLATULENCE	6 (2.0)	6 (3.2)	3 (1.6)
ABDOMINAL PAIN UPPER	4 (1.3)	3 (1.6)	3 (1.6)
DYSPEPSIA	4 (1.3)	4 (2.1)	1 (0.5)
ABDOMINAL DISTENSION	2 (0.7)	2 (1.1)	1 (0.5)
DRY MOUTH	2 (0.7)	1 (0.5)	1 (0.5)
HAEMATOCHEZIA	2 (0.7)	2 (1.1)	0
HAEMORRHOIDS	2 (0.7)	2 (1.1)	0
COLITIS	1 (0.3)	0	0
COLITIS ISCHAEMIC	1 (0.3)	1 (0.5)	0
DAEFECATION URGENCY	1 (0.3)	1 (0.5)	0
DYSPHAGIA	1 (0.3)	1 (0.5)	0
ENTERITIS	1 (0.3)	1 (0.5)	0
FAECES SOFT	1 (0.3)	0	0

Note: Adverse events are coded using MedDRA version 17.0. A subject is counted once within each system organ class and preferred term.
[1] Includes Study KRX-0502-306 and Study KRX-0502-204 double-blind KRX-0502, Study KRX-0502-306 open-label extension period, and open-label Study KRX-0502-207.
[2] TEAEs are defined as adverse events occurring or worsening in severity after the first dose of study drug. GI events of interest include all adverse events under the MedDRA System Organ Class of Gastrointestinal Disorders.

Table 3.22.1
Number and Percent of Subjects with Treatment-Emergent Gastrointestinal (GI) Adverse Events
by System Organ Class and Preferred Term
Pooled Safety Population

System Organ Class<br>Preferred Term	All<br>KRX-0502 [1]<br>(N=301)<br>n(%)	Double-Blind<br>KRX-0502<br>(N=190)<br>n(%)	Double-Blind<br>Placebo<br>(N=188)<br>n(%)
GASTROINTESTINAL DISORDERS (cont)			
FOOD POISONING	1 (0.3)	1 (0.5)	0
FREQUENT BOWEL MOVEMENTS	1 (0.3)	1 (0.5)	0
GASTROINTESTINAL HAEMORRHAGE	1 (0.3)	0	0
GASTROOESOPHAGEAL REFLUX DISEASE	1 (0.3)	0	3 (1.6)
MUCOUS STOOLS	1 (0.3)	1 (0.5)	0
RECTAL HAEMORRHAGE	1 (0.3)	1 (0.5)	0
TOOTHACHE	1 (0.3)	1 (0.5)	1 (0.5)
UMBILICAL HERNIA	1 (0.3)	1 (0.5)	0
VOMITING PROJECTILE	1 (0.3)	1 (0.5)	0
FAECAL INCONTINENCE	0	0	1 (0.5)
FAECES HARD	0	0	1 (0.5)
GASTRITIS	0	0	1 (0.5)
PANCREATITIS	0	0	1 (0.5)

Note: Adverse events are coded using MedDRA version 17.0. A subject is counted once within each system organ class and preferred term.
[1] Includes Study KRX-0502-306 and Study KRX-0502-204 double-blind KRX-0502, Study KRX-0502-306 open-label extension period, and open-label Study KRX-0502-207.
[2] TEAEs are defined as adverse events occurring or worsening in severity after the first dose of study drug. GI events of interest include all adverse events under the MedDRA System Organ Class of Gastrointestinal Disorders.
