# Supplementary material for: Safety and efficacy of ferric citrate in patients with nondialysis-dependent chronic kidney disease
Source: PLoS One. 2017 Nov 29;12(11):e0188712. doi: 10.1371/journal.pone.0188712 (PMC5706696; doi:10.1371/journal.pone.0188712)
Supplement: S2 Table — (PDF) [file pone.0188712.s002.pdf]

Table 3.12
Number and Percent of Subjects with Treatment-Emergent Adverse Events
by System Organ Class, Preferred Term, and Time to Onset
Pooled Safety Population

System Organ Class<br>Preferred Term	Time to Onset<br>(weeks)	All<br>KRX-0502 [1]<br>(N=301)<br>n(%)	Double-Blind<br>KRX-0502<br>(N=190)<br>n(%)	Double-Blind<br>Placebo<br>(N=188)<br>n(%)
GASTROINTESTINAL DISORDERS	>0 - 4	98 (32.6)	64 (33.7)	30 (16.0)
	>4 - 8	42 (15.0)	32 (18.2)	18 (11.1)
	>8 - 12	13 (6.5)	12 (7.3)	8 (5.3)
	>12 - 16	14 (10.6)	14 (10.5)	8 (6.3)
	>16 - 20	6 (6.9)	0	0
	>20	5 (6.0)	0	0
DIARRHOEA	>0 - 4	34 (11.3)	24 (12.6)	13 (6.9)
	>4 - 8	17 (6.1)	11 (6.3)	6 (3.7)
	>8 - 12	6 (3.0)	5 (3.0)	4 (2.7)
	>12 - 16	6 (4.5)	6 (4.5)	2 (1.6)
	>16 - 20	3 (3.4)	0	0
	>20	0	0	0
FAECES DISCOLOURED	>0 - 4	52 (17.3)	35 (18.4)	0
	>4 - 8	3 (1.1)	2 (1.1)	0
	>8 - 12	3 (1.5)	3 (1.8)	0
	>12 - 16	1 (0.8)	1 (0.8)	0
	>16 - 20	0	0	0
	>20	0	0	0

Note: Adverse events are coded using MedDRA version 17.0. For each system organ class and preferred term, a subject is counted once within each of the intervals in which the event occurred. The number of subjects treated in the interval is the denominator for the percentages.

[1] Includes Study KRX-0502-306 and Study KRX-0502-204 double-blind KRX-0502, Study KRX-0502-306 open-label extension period, and open-label Study KRX-0502-207.

[2] TEAEs are defined as adverse events occurring or worsening in severity after the first dose of study drug.
