# Supplementary material for: Safety and efficacy of ferric citrate in patients with nondialysis-dependent chronic kidney disease
Source: PLoS One. 2017 Nov 29;12(11):e0188712. doi: 10.1371/journal.pone.0188712 (PMC5706696; doi:10.1371/journal.pone.0188712)
Supplement: S3 Table — (PDF) [file pone.0188712.s003.pdf]

Table 1.1
Mixed Model Repeated Measures (MMRM) of Change in Hemoglobin (g/dL) from Baseline over Time During Double-Blind Period
Studies KRX-0502-306 and KRX-0502-204

	KRX-0502	Placebo	Difference (KRX-0502 - Placebo)
Week 1 Change from Baseline			
n	180	171	
LS Mean (SE)	-0.05 (0.055)	-0.12 (0.056)	0.07 (0.077)
95% Confidence Interval	(-0.154, 0.061)	(-0.227, -0.007)	(-0.081, 0.223)
Treatment p-value			0.361
Week 2 Change from Baseline			
n	180	164	
LS Mean (SE)	0.05 (0.055)	-0.17 (0.057)	0.22 (0.078)
95% Confidence Interval	(-0.058, 0.157)	(-0.284, -0.061)	(0.068, 0.375)
Treatment p-value			0.005
Week 4 Change from Baseline			
n	171	158	
LS Mean (SE)	0.19 (0.056)	-0.19 (0.057)	0.37 (0.079)
95% Confidence Interval	(0.076, 0.295)	(-0.300, -0.074)	(0.217, 0.528)
Treatment p-value			<0.001
Week 6 Change from Baseline			
n	160	151	
LS Mean (SE)	0.27 (0.057)	-0.18 (0.058)	0.45 (0.081)
95% Confidence Interval	(0.163, 0.386)	(-0.293, -0.065)	(0.295, 0.612)
Treatment p-value			<0.001

Note: By visit results are from a mixed model repeated measures (MMRM) analysis using restricted maximum likelihood (REML) with treatment (KRX-0502 versus placebo), study, visit, and treatment-by-visit interaction as fixed effects, baseline as a covariate, and subject as a random effect. Study-by-treatment interaction p-value was obtained from the same model with the addition of the study-by-treatment interaction term.

Table 1.1
Mixed Model Repeated Measures (MMRM) of Change in Hemoglobin (g/dL) from Baseline over Time During Double-Blind Period
Studies KRX-0502-306 and KRX-0502-204

	KRX-0502	Placebo	Difference (KRX-0502 - Placebo)
Week 8 Change from Baseline			
n	156	140	
LS Mean (SE)	0.41 (0.057)	-0.14 (0.060)	0.55 (0.082)
95% Confidence Interval	(0.298, 0.523)	(-0.261, -0.027)	(0.394, 0.715)
Treatment p-value			<0.001
Week 10 Change from Baseline			
n	152	137	
LS Mean (SE)	0.47 (0.058)	-0.18 (0.060)	0.65 (0.083)
95% Confidence Interval	(0.359, 0.586)	(-0.297, -0.062)	(0.490, 0.814)
Treatment p-value			<0.001
Week 12 Change from Baseline			
n	158	150	
LS Mean (SE)	0.50 (0.057)	-0.17 (0.058)	0.67 (0.081)
95% Confidence Interval	(0.389, 0.612)	(-0.284, -0.055)	(0.512, 0.829)
Treatment p-value			<0.001
Week 14 Change from Baseline			
n	91	86	
LS Mean (SE)	0.63 (0.068)	-0.16 (0.070)	0.79 (0.097)
95% Confidence Interval	(0.499, 0.767)	(-0.298, -0.023)	(0.603, 0.983)
Treatment p-value			<0.001

Note: By visit results are from a mixed model repeated measures (MMRM) analysis using restricted maximum likelihood (REML) with treatment (KRX-0502 versus placebo), study, visit, and treatment-by-visit interaction as fixed effects, baseline as a covariate, and subject as a random effect. Study-by-treatment interaction p-value was obtained from the same model with the addition of the study-by-treatment interaction term.

Table 1.1
Mixed Model Repeated Measures (MMRM) of Change in Hemoglobin (g/dL) from Baseline over Time During Double-Blind Period
Studies KRX-0502-306 and KRX-0502-204

	KRX-0502	Placebo	Difference (KRX-0502 - Placebo)
Week 16 Change from Baseline			
n	86	77	
LS Mean (SE)	0.77 (0.070)	-0.08 (0.073)	0.86 (0.100)
95% Confidence Interval	(0.637, 0.911)	(-0.224, 0.062)	(0.660, 1.051)
Treatment p-value			<0.001

Study-by-treatment interaction p-value = 0.624

Note: By visit results are from a mixed model repeated measures (MMRM) analysis using restricted maximum likelihood (REML) with treatment (KRX-0502 versus placebo), study, visit, and treatment-by-visit interaction as fixed effects, baseline as a covariate, and subject as a random effect. Study-by-treatment interaction p-value was obtained from the same model with the addition of the study-by-treatment interaction term.
