# Supplementary material for: Safety and efficacy of ferric citrate in patients with nondialysis-dependent chronic kidney disease
Source: PLoS One. 2017 Nov 29;12(11):e0188712. doi: 10.1371/journal.pone.0188712 (PMC5706696; doi:10.1371/journal.pone.0188712)
Supplement: S4 Table — (PDF) [file pone.0188712.s004.pdf]

Table 1.2
Mixed Model Repeated Measures (MMRM) of Change in Iron Study Parameters from Baseline over Time During Double-Blind Period
Studies KRX-0502-306 and KRX-0502-204

Transferrin Saturation (%)

	KRX-0502	Placebo	Difference (KRX-0502 - Placebo)
Week 1 Change from Baseline			
n	176	171	
LS Mean (SE)	3.78 (0.758)	-1.33 (0.768)	5.11 (1.071)
95% Confidence Interval	(2.298, 5.270)	(-2.832, 0.180)	(3.009, 7.212)
Treatment p-value			<0.001
Week 2 Change from Baseline			
n	179	161	
LS Mean (SE)	5.39 (0.752)	-0.89 (0.789)	6.28 (1.083)
95% Confidence Interval	(3.910, 6.861)	(-2.439, 0.654)	(4.155, 8.401)
Treatment p-value			<0.001
Week 4 Change from Baseline			
n	170	158	
LS Mean (SE)	5.96 (0.770)	-1.27 (0.796)	7.23 (1.100)
95% Confidence Interval	(4.449, 7.470)	(-2.828, 0.295)	(5.069, 9.384)
Treatment p-value			<0.001
Week 6 Change from Baseline			
n	159	147	
LS Mean (SE)	7.04 (0.792)	-1.75 (0.820)	8.79 (1.133)
95% Confidence Interval	(5.482, 8.588)	(-3.359, -0.142)	(6.563, 11.008)
Treatment p-value			<0.001

Note: By visit results are from a mixed model repeated measures (MMRM) analysis using restricted maximum likelihood (REML) with treatment (KRX-0502 versus placebo), study, visit, and treatment-by-visit interaction as fixed effects, baseline as a covariate, and subject as a random effect. Study-by-treatment interaction p-value was obtained from the same model with the addition of the study-by-treatment interaction term.

Table 1.2
Mixed Model Repeated Measures (MMRM) of Change in Iron Study Parameters from Baseline over Time During Double-Blind Period
Studies KRX-0502-306 and KRX-0502-204

Transferrin Saturation (%)

	KRX-0502	Placebo	Difference (KRX-0502 - Placebo)
Week 8 Change from Baseline			
n	155	140	
LS Mean (SE)	9.29 (0.800)	-1.39 (0.838)	10.69 (1.152)
95% Confidence Interval	(7.725, 10.864)	(-3.036, 0.252)	(8.427, 12.945)
Treatment p-value			<0.001
Week 10 Change from Baseline			
n	147	138	
LS Mean (SE)	12.33 (0.817)	-1.25 (0.843)	13.57 (1.168)
95% Confidence Interval	(10.723, 13.929)	(-2.901, 0.406)	(11.283, 15.863)
Treatment p-value			<0.001
Week 12 Change from Baseline			
n	156	151	
LS Mean (SE)	12.28 (0.796)	-1.56 (0.808)	13.84 (1.130)
95% Confidence Interval	(10.721, 13.842)	(-3.140, 0.030)	(11.621, 16.053)
Treatment p-value			<0.001
Week 14 Change from Baseline			
n	90	86	
LS Mean (SE)	15.14 (1.016)	-1.49 (1.039)	16.63 (1.436)
95% Confidence Interval	(13.145, 17.130)	(-3.527, 0.548)	(13.811, 19.443)
Treatment p-value			<0.001

Note: By visit results are from a mixed model repeated measures (MMRM) analysis using restricted maximum likelihood (REML) with treatment (KRX-0502 versus placebo), study, visit, and treatment-by-visit interaction as fixed effects, baseline as a covariate, and subject as a random effect. Study-by-treatment interaction p-value was obtained from the same model with the addition of the study-by-treatment interaction term.

Table 1.2
Mixed Model Repeated Measures (MMRM) of Change in Iron Study Parameters from Baseline over Time During Double-Blind Period
Studies KRX-0502-306 and KRX-0502-204

Transferrin Saturation (%)

	KRX-0502	Placebo	Difference (KRX-0502 - Placebo)
Week 16 Change from Baseline			
n	79	80	
LS Mean (SE)	15.76 (1.075)	-0.44 (1.072)	16.20 (1.502)
95% Confidence Interval	(13.650, 17.866)	(-2.540, 1.664)	(13.252, 19.141)
Treatment p-value			<0.001

Study-by-treatment interaction p-value = 0.055

Note: By visit results are from a mixed model repeated measures (MMRM) analysis using restricted maximum likelihood (REML) with treatment (KRX-0502 versus placebo), study, visit, and treatment-by-visit interaction as fixed effects, baseline as a covariate, and subject as a random effect. Study-by-treatment interaction p-value was obtained from the same model with the addition of the study-by-treatment interaction term.

Table 1.2
Mixed Model Repeated Measures (MMRM) of Change in Iron Study Parameters from Baseline over Time During Double-Blind Period
Studies KRX-0502-306 and KRX-0502-204

Ferritin (ng/mL)

	KRX-0502	Placebo	Difference (KRX-0502 - Placebo)
Week 1 Change from Baseline			
n	178	172	
LS Mean (SE)	3.91 (3.617)	-3.27 (3.669)	7.18 (5.098)
95% Confidence Interval	(-3.189, 11.006)	(-10.471, 3.927)	(-2.821, 17.182)
Treatment p-value			0.159
Week 2 Change from Baseline			
n	180	163	
LS Mean (SE)	10.41 (3.606)	-3.99 (3.742)	14.39 (5.143)
95% Confidence Interval	(3.330, 17.481)	(-11.328, 3.355)	(4.302, 24.482)
Treatment p-value			0.005
Week 4 Change from Baseline			
n	171	158	
LS Mean (SE)	19.62 (3.672)	-2.13 (3.788)	21.75 (5.223)
95% Confidence Interval	(12.414, 26.824)	(-9.559, 5.303)	(11.500, 31.994)
Treatment p-value			<0.001
Week 6 Change from Baseline			
n	162	152	
LS Mean (SE)	32.57 (3.739)	-6.29 (3.836)	38.86 (5.306)
95% Confidence Interval	(25.240, 39.910)	(-13.814, 1.239)	(28.453, 49.272)
Treatment p-value			<0.001

Note: By visit results are from a mixed model repeated measures (MMRM) analysis using restricted maximum likelihood (REML) with treatment (KRX-0502 versus placebo), study, visit, and treatment-by-visit interaction as fixed effects, baseline as a covariate, and subject as a random effect. Study-by-treatment interaction p-value was obtained from the same model with the addition of the study-by-treatment interaction term.

Table 1.2
Mixed Model Repeated Measures (MMRM) of Change in Iron Study Parameters from Baseline over Time During Double-Blind Period
Studies KRX-0502-306 and KRX-0502-204

Ferritin (ng/mL)

	KRX-0502	Placebo	Difference (KRX-0502 - Placebo)
Week 8 Change from Baseline			
n	156	141	
LS Mean (SE)	45.40 (3.785)	-8.09 (3.935)	53.49 (5.409)
95% Confidence Interval	(37.975, 52.825)	(-15.805, -0.366)	(42.876, 64.097)
Treatment p-value			<0.001
Week 10 Change from Baseline			
n	152	138	
LS Mean (SE)	69.93 (3.816)	-6.45 (3.962)	76.38 (5.451)
95% Confidence Interval	(62.441, 77.414)	(-14.221, 1.322)	(65.684, 87.071)
Treatment p-value			<0.001
Week 12 Change from Baseline			
n	159	151	
LS Mean (SE)	93.79 (3.751)	-5.87 (3.826)	99.66 (5.321)
95% Confidence Interval	(86.432, 101.152)	(-13.377, 1.637)	(89.224, 110.100)
Treatment p-value			<0.001
Week 14 Change from Baseline			
n	92	86	
LS Mean (SE)	116.23 (4.561)	-9.72 (4.696)	125.94 (6.468)
95% Confidence Interval	(107.285, 125.174)	(-18.925, -0.505)	(113.260, 138.630)
Treatment p-value			<0.001

Note: By visit results are from a mixed model repeated measures (MMRM) analysis using restricted maximum likelihood (REML) with treatment (KRX-0502 versus placebo), study, visit, and treatment-by-visit interaction as fixed effects, baseline as a covariate, and subject as a random effect. Study-by-treatment interaction p-value was obtained from the same model with the addition of the study-by-treatment interaction term.

Table 1.2
Mixed Model Repeated Measures (MMRM) of Change in Iron Study Parameters from Baseline over Time During Double-Blind Period
Studies KRX-0502-306 and KRX-0502-204

Ferritin (ng/mL)

	KRX-0502	Placebo	Difference (KRX-0502 - Placebo)
Week 16 Change from Baseline			
n	86	81	
LS Mean (SE)	148.14 (4.673)	-7.94 (4.800)	156.08 (6.623)
95% Confidence Interval	(138.973, 157.304)	(-17.351, 1.476)	(143.088, 169.065)
Treatment p-value			<0.001

Study-by-treatment interaction p-value = 0.076

Note: By visit results are from a mixed model repeated measures (MMRM) analysis using restricted maximum likelihood (REML) with treatment (KRX-0502 versus placebo), study, visit, and treatment-by-visit interaction as fixed effects, baseline as a covariate, and subject as a random effect. Study-by-treatment interaction p-value was obtained from the same model with the addition of the study-by-treatment interaction term.
