# Supplementary material for: Safety and efficacy of ferric citrate in patients with nondialysis-dependent chronic kidney disease
Source: PLoS One. 2017 Nov 29;12(11):e0188712. doi: 10.1371/journal.pone.0188712 (PMC5706696; doi:10.1371/journal.pone.0188712)
Supplement: S5 Table — (PDF) [file pone.0188712.s005.pdf]

WCN Abstract

Mixed Model Repeated Measures (MMRM) of Change in Serum Chemistry Parameters from Baseline over Time During Double-Blind Period

Studies KRX-0502-306 and KRX-0502-204

Phosphate (mg/dL)

	KRX-0502	Placebo	Difference (KRX-0502 - Placebo)
Week 1 Change from Baseline			
n	180	172	
LS Mean (SE)	-0.25 (0.044)	-0.08 (0.045)	-0.17 (0.062)
95% Confidence Interval	(-0.336, -0.165)	(-0.167, 0.008)	(-0.292, -0.050)
Treatment p-value			0.006
Week 2 Change from Baseline			
n	181	165	
LS Mean (SE)	-0.28 (0.044)	-0.05 (0.045)	-0.22 (0.062)
95% Confidence Interval	(-0.361, -0.190)	(-0.144, 0.034)	(-0.343, -0.098)
Treatment p-value			<0.001
Week 4 Change from Baseline			
n	172	158	
LS Mean (SE)	-0.31 (0.045)	-0.05 (0.046)	-0.27 (0.064)
95% Confidence Interval	(-0.402, -0.227)	(-0.139, 0.042)	(-0.391, -0.141)
Treatment p-value			<0.001
Week 6 Change from Baseline			
n	162	151	
LS Mean (SE)	-0.35 (0.046)	-0.19 (0.047)	-0.17 (0.065)
95% Confidence Interval	(-0.441, -0.262)	(-0.277, -0.093)	(-0.294, -0.039)
Treatment p-value			0.011

Note: By visit results are from a mixed model repeated measures (MMRM) analysis using restricted maximum likelihood (REML) with treatment (KRX-0502 versus placebo), study, visit, and treatment-by-visit interaction as fixed effects, baseline as a covariate, and subject as a random effect. Study-by-treatment interaction p-value was obtained from the same model with the addition of the study-by-treatment interaction term.

WCN Abstract

Mixed Model Repeated Measures (MMRM) of Change in Serum Chemistry Parameters from Baseline over Time During Double-Blind Period

Studies KRX-0502-306 and KRX-0502-204

Phosphate (mg/dL)

	KRX-0502	Placebo	Difference (KRX-0502 - Placebo)
Week 8 Change from Baseline			
n	156	141	
LS Mean (SE)	-0.38 (0.046)	-0.18 (0.048)	-0.21 (0.066)
95% Confidence Interval	(-0.474, -0.292)	(-0.273, -0.083)	(-0.335, -0.075)
Treatment p-value			0.002
Week 10 Change from Baseline			
n	151	138	
LS Mean (SE)	-0.42 (0.047)	-0.15 (0.049)	-0.28 (0.067)
95% Confidence Interval	(-0.514, -0.330)	(-0.242, -0.051)	(-0.407, -0.144)
Treatment p-value			<0.001
Week 12 Change from Baseline			
n	159	151	
LS Mean (SE)	-0.42 (0.046)	-0.15 (0.047)	-0.27 (0.065)
95% Confidence Interval	(-0.514, -0.335)	(-0.246, -0.063)	(-0.398, -0.142)
Treatment p-value			<0.001
Week 14 Change from Baseline			
n	92	86	
LS Mean (SE)	-0.48 (0.057)	-0.18 (0.059)	-0.31 (0.082)
95% Confidence Interval	(-0.597, -0.372)	(-0.293, -0.060)	(-0.468, -0.148)
Treatment p-value			<0.001

Note: By visit results are from a mixed model repeated measures (MMRM) analysis using restricted maximum likelihood (REML) with treatment (KRX-0502 versus placebo), study, visit, and treatment-by-visit interaction as fixed effects, baseline as a covariate, and subject as a random effect. Study-by-treatment interaction p-value was obtained from the same model with the addition of the study-by-treatment interaction term.

WCN Abstract
Mixed Model Repeated Measures (MMRM) of Change in Serum Chemistry Parameters from Baseline over Time During Double-Blind Period
Studies KRX-0502-306 and KRX-0502-204

Phosphate (mg/dL)

	KRX-0502	Placebo	Difference (KRX-0502 - Placebo)
Week 16 Change from Baseline			
n	86	81	
LS Mean (SE)	-0.52 (0.059)	-0.22 (0.061)	-0.30 (0.084)
95% Confidence Interval	(-0.637, -0.405)	(-0.341, -0.102)	(-0.464, -0.135)
Treatment p-value			<0.001

Study-by-treatment interaction p-value = <.001

Note: By visit results are from a mixed model repeated measures (MMRM) analysis using restricted maximum likelihood (REML) with treatment (KRX-0502 versus placebo), study, visit, and treatment-by-visit interaction as fixed effects, baseline as a covariate, and subject as a random effect. Study-by-treatment interaction p-value was obtained from the same model with the addition of the study-by-treatment interaction term.
